# Identification and Spatial Visualization of Dysregulated Bile Acid Metabolism in High-Fat Diet-Fed Mice by Mass Spectral Imaging

**DOI:** 10.3389/fnut.2022.858603

**Published:** 2022-03-30

**Authors:** Qi Zhang, Zhen-Hua Wu, Shan-Shan Zhao, Jing Yang, Lei Chen, Xiao-Yu Wang, Zhan-You Wang, Hui-Xin Liu

**Affiliations:** ^1^Health Sciences Institute, China Medical University, Shenyang, China; ^2^Institute of Life Sciences, China Medical University, Shenyang, China; ^3^Liaoning Key Laboratory of Obesity and Glucose/Lipid Associated Metabolic Diseases, China Medical University, Shenyang, China

**Keywords:** mass spectrometry imaging (MSI), MALDI, bile acid, zonation pattern, enterohepatic circulation, metabolic disease

## Abstract

Changes in overall bile acid (BA) levels and specific BA metabolites are involved in metabolic diseases, gastrointestinal, and liver cancer. BAs have become established as important signaling molecules that enable fine-tuned inter-tissue communication within the enterohepatic circulation. The liver, BAs site of production, displayed physiological and functional zonal differences in the periportal zone versus the centrilobular zone. In addition, BA metabolism shows regional differences in the intestinal tract. However, there is no available method to detect the spatial distribution and molecular profiling of BAs within the enterohepatic circulation. Herein, we demonstrated the application in mass spectrometry imaging (MSI) with a high spatial resolution (3 μm) plus mass accuracy matrix-assisted laser desorption ionization (MALDI) to imaging BAs and *N*-1-naphthylphthalamic acid (NPA). Our results could clearly determine the zonation patterns and regional difference characteristics of BAs on mouse liver, ileum, and colon tissue sections, and the relative content of BAs based on NPA could also be ascertained. In conclusion, our method promoted the accessibility of spatial localization and quantitative study of BAs on gastrointestinal tissue sections and demonstrated that MALDI-MSI was a valuable tool to investigate and locate several BA molecules in different tissue types leading to a better understanding of the role of BAs behind the gastrointestinal diseases.

## Graphical Abstract

Schematic illustrations showing the mass spectrometry imaging based identification and spatial visualization of dysregulated bile acid metabolism in high-fat diet-fed mice.

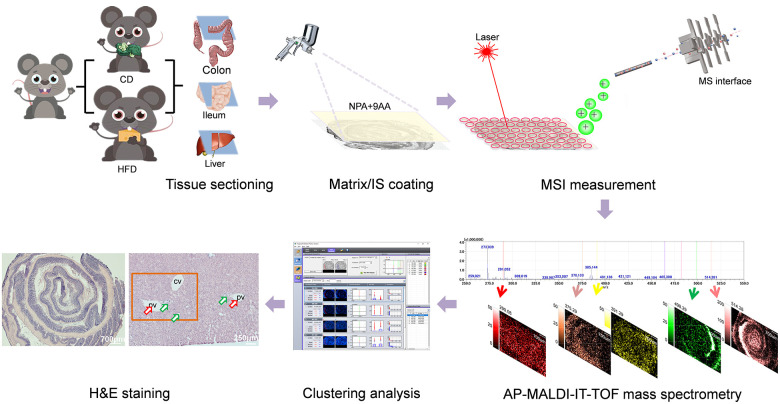

## Introduction

The liver and gut share an intimate relationship whose communication relies heavily on metabolites, among which bile acids (BAs) play a key role (1). Previous work from our lab and others demonstrated that BAs play a regulatory role in host metabolism and immune responses (1–5). Changes to the composition and distribution of the BAs have been implicated in the etiopathogenesis of multiple diseases ranging from the liver to the intestine (6). Liver diseases differentially affect BA concentration and distribution and composition, so as in the intestine (7). Strong imbalance or disruption in BA synthesis and secretion processes are associated with various liver and gastrointestinal diseases, such as BA synthesis disorder (inborn) and primary biliary cholangitis (8). Recent studies demonstrated that microbial BA metabolism and its alterations by diet and host signaling are implicated in obesity associated metabolic diseases (9). However, the information of the abundance and spatial distribution of BAs and link them to the pathology of the corresponding diseases are still elusive.

Bile acids, whose functional role in the global mammalian system is multifaceted, are an important class of metabolites that undergo extensive enterohepatic recycling and gut microbial modification (10). To understand complex BA processes and disorders and develop effective treatments, it is important to have reliable information on the abundance and spatial distribution of BAs and their interplay with their receptors and link them to the pathology of the corresponding diseases. Because of the great diversity of BAs and the complexity of their signaling mechanisms, it is very useful to image several BAs in a single analysis in a way that enables simultaneous measurement and characterization of numerous BAs. However, simultaneous spatial mapping and quantitation of changes of BAs in enterohepatic circulation represent a significant challenge. Over the last few decades, mass spectrometry (MS)-based techniques for the detection of BAs are mostly indirect assays for detecting the content of BAs in tissue homogenate or the spatial distribution of BAs in tissues without quantification (11–14). In addition, benchmark methods for BA analysis, such as liquid chromatography (LC) and electrospray ionization (ESI)-MS, require extensive processing of liver or intestine tissue samples before analysis, which causes losses of anatomical information (11, 15, 16). Thus, there is a need for innovative imaging techniques that can simultaneously map and quantitate the BAs in tissue samples. However, mass spectrometry imaging (MSI) of BAs in the organs is challenging because of their poor ionization properties and the highly ion-suppressing tissue microenvironment. Despite the difficulties of imaging BAs and their metabolites in the colon and other organs, efforts have been made using commercially available or in-house constructed ion sources. Matrix assisted laser desorption ionization (MALDI)’s high tolerance to contaminants and simplicity of preparation make it ideal for the analysis of BAs from biological media. Recently, a novel imaging mass microscope (iMScope) shows promise in simultaneously assessing the spatial distribution and molecular profiling in a non-targeted manner (17, 18).

Thus, in our study, the iMScope was adapted to investigate the spatial distribution and molecular profiling of BAs in the liver and intestine slices of a mouse under high-fat diet (HFD) to indicate the effects of metabolism of BAs on the health and diseases of the body.

## Methods

### Chemicals

Mass spectrometry grade chemicals and reagents were used in the present study. Matrix moieties inclusive of 9-aminoacridine (9AA, no. 92817), 2, 5-dihydroxybenzoic acid (DHB, no. 149357), α-cyano-4-hydroxycinnamic acid (CHCA, no. C2020) were obtained from Sigma-Aldrich (St Louis, MO, United States). The internal standard (IS), *N*-1-naphthylphthalamic acid (NPA), was purchased from Chem Service (West Chester, PA, United States, no. N875498). Ultra-pure grade (18 MU) water was prepared by a Milli-Q system (Millipore Corporation, Billerica, MA, United States). Other reagents include formic acid (FA; Aladdin, no. F112034); Trifluoroacetic Acid (TFA; TEDIA, no. TS4295-013C); Methanol (MeOH; Honeywell, no. AH230-4HC); Ethanol (EtOH; Sigma-Aldrich, no. E7023); Ethanol (Tianjin Fuyu Fine Chemical Co., Ltd.); Acetonitrile (ACN; Honeywell, No. AH015-4HC); Xylene (Tianjin Fuyu Fine Chemical Co., Ltd.); Eosin (Beijing Solarbio Technology Co., Ltd., cat. no. G1100); Neutral gum (Beijing Solarbio Technology Co., Ltd., cat. no. G8590-100 ml), and indium tin oxide (ITO)-coated glass slides (Sigma-Aldrich, no. 578274).

Eleven BAs were selected for analysis: deoxycholic acid (DCA), chenodeoxycholic acid (CDCA), ursodeoxycholic acid (UDCA), taurodeoxycholic acid (TDCA), taurochenodeoxycholic acid (TCDCA), tauroursodeoxycholic acid (TUDCA), lithocholic acid (LCA), taurolithocholic acid (TLCA), taurocholic acid (TCA), tauromuricholic acid (TMCA), and glycocholic acid (GCA).

### Animal Study

All animal procedures were approved by the Institutional Animal Care and Use Committee (IACUC) at the China Medical University. Six to eight weeks old male C57BL/6 mice were purchased from Beijing Huafukang Biotechnology Co., Ltd. The mice were fed with normal circadian circulation for 12 h and had enough food and water. Mice were fed an HFD (TP23520, Tropic diet, China) or a control diet (CD, TP23524, Tropic diet, China) for 4 weeks. There were 6–10 mice in both the CD group and the HFD group. After 4 weeks of feeding, animals were sacrificed for analysis.

### Sample Preparation

After euthanasia, the tissues were quickly removed and all samples should be frozen immediately and stored at −80°C until further analysis. Set the cryomicrotome (Leica CM1950, Nussloch, Germany) to −25°C and cut the samples at a thickness of 10 μm. The slices were pasted on ITO-coated glass slide for analysis by MALDI-MSI. Three to five serial sections (each 10 μm thick) of sampled liver, ileum, and colon tissue were used to evaluate the reproducibility of the iMScope technique. A small droplet of optimal cutting temperature (OCT) compound is applied for mounting the tissue sample on the specimen holder. Because polyvinyl alcohol/polyethylene glycol-based media, such as OCT compound, cause ion suppression during MSI analysis, it is essential to avoid contamination of tissue sections, cryostat tables, or blades by OCT compounds (19). Subsequently, a “two-step matrix application,” which combined with sublimation and airbrushing, was used to coat matrix for tissue sections (18, 20). (1) Sublimation: the electrically conductive glass slide bearing specimen (slice spiked with 10 ng ml^–1^ of NPA) was installed in a sample holder, which was then embedded in a vacuum deposition system (SVC-700TMSG iMLayer, Sanyu Electron, Tokyo, Japan). A matrix holder was filled with approximately 300 mg of matrix powder (9AA) and the sample holder and matrix bracket were positioned with 8 cm distance. The matrix power was then heated to the boiling point of the matrix crystals (220°C for 9AA) and the vapor covered the specimen surface for 8 min. The vacuum pressure of the chamber was maintained at 10^–4^ Pa in the process of sublimation. (2) Airbrushing: Matrix solution (10 mg ml^–1^ of 9AA) was prepared by dissolving matrix power in acetonitrile and distilled water (all containing 0.1% FA) at a ratio of 1:1. The matrix solution (1 ml) was added to the capacity of an artist’s airbrush (MR. Linear Compressor L7/PS270 Airbrush, GSI Creos, Tokyo, Japan). The distance between the tip of the airbrush and the tissue surface was about 8 cm. For the first 3 cycles, the matrix was airbrushed for 2 s at 60 s intervals and, in the following 20 cycles, the matrix was continuously sprayed for 1.0 s at 30 s intervals. The glass slide was then placed in a vacuum dryer to vaporize the solvent for 5 min. After that, the glass slide was vacuum-dried, and then the BA distribution was observed with iMScope.

### Matrix-Assisted Laser Desorption-Ionization Time-of-Flight-Mass Spectrometry

The parameters of IT-TOF (time-of-flight) MS were set as follows: ion polarity, negative; mass range, 350–550; sample voltage, 3.0 kV; and detector voltage, 1.90 kV. The imaging MS Solution Version 1.12.26 software (Shimadzu, Tokyo, Japan) was used to control the instrument, and the data acquisition, visualization, and quantification were also performed by the same software. The *m/z* values were externally calibrated using the DHB matrix. The identification of BAs was confirmed by MALDI-TOF-MS/MS with reference to product ion spectra of authentic BA standards.

### Visualization of Bile Acids Distribution in Mice Tissue by Novel Imaging Mass Microscope

The iMScope TRIO (Shimadzu, Japan) instrument, a hybrid IT-TOF MS combining an optical microscope and atmospheric pressure MALDI/ionization system, was used to acquire the imaging MS data. One of the most critical processes in MSI is the creation of the region of interest (ROI). The optical microscope embedded in the iMScope permitted us to precisely choose the relevant tissue region prior to performing data acquisition. An ultraviolet laser, tightly focused with a triplet lens, was used to ensure high spatial resolution. Based on the advanced configuration above, a tissue ROI was freely selected *via* a charge-coupled device (CCD) camera (magnification, ×1.25/×2.5, Olympus Corporation, Tokyo, Japan) and the imaging area was then defined according to the maximum imaging point under a scan pitch of 40 μm. The illumination in the iMScope was operated under the following parameters: light type, *trans*-illumination; light intensity, 12%. Foci and observation points were controlled with a joystick and the XYZ stage (Kohzu Precision, Kanagawa, Japan) on which the electrically conductive glass slide was fixed. The XYZ coordinates, with position-feedback scales, immediately displayed the ROIs to make position reproducibility possible on a sub-micrometer order. The tissue slices were then irradiated by a focused laser beam in synchrony with stage scanning. The laser in the iMScope system was a diode-pumped 355 nm Nd, YAG laser (Shimadzu Corporation, Kyoto, Japan), and operated under the following parameters: frequency, 1,000 Hz; laser intensity, 55.0; and laser diameter, 3 μm. All the experiments in this work were conducted with the minimum irradiation diameter and irradiated the tissue surface with 100 shots (repetition rate; 1,000 Hz) for each pixel.

### Selection of Internal Standard and Semi-Quantitative of Bile Acids

Quantitative analysis with MALDI-TOF MS has been demonstrated for compounds of biological interest. For quantification, ISs are necessary to compensate for the poor shot-to-shot reproducibility inherent in the use of MALDI analysis. An ideal IS should be the stable isotope-labeled intact BAs during the qualitative and quantitative analyses of BAs (21). However, it was impractical in the present case due to synthesis challenges and time vs. cost concerns. Therefore, we want to search for some compounds that are chemically similar to BAs, have a quality close to BAs, and are chemically stable during the analysis process as ISs. Unfortunately, we did not find it either. While the NPA chosen for this analysis is chemically different from BAs, it still proved to be effective. NPA has been used to quantify BAs directly from plasma and urine by MALDI-TOF-MS (22, 23). The structure and MS spectrum of NPA are shown in Supplementary Figure 1. We used the average intensity ratio of each BA to NPA to quantitatively describe the relative exposure level of BAs in tissue sections. The average peak intensity ratio was calculated as follows: average peak intensity ratio = BAs average peak intensity/NPA average peak intensity.

### Tissue Preparation for Histology

After MSI, the tissue sections were stained with hematoxylin and eosin (H&E) for examination based on a protocol (11). In brief, the tissue sections were stained with H&E as follows: the glass slides were gently washed with 70% ethanol to remove the matrix, followed by dipped in 100% ethanol at −20°C for 5 min, and then glass slides were put in distilled water for 2 min. The slides were then immersed in hematoxylin for 5 min, washed with tap water for 5 min, followed by differentiated with 1% hydrochloric acid alcohol for 5 s, and blued in running tap water for 10 min. Staining by eosin for 5 s was followed by a wash with tap water for 5 min. Slides were then dipped in 90% ethanol for 6 min, and 100% ethanol for 2 × 5 min, followed by placing them in three different xylene baths for 5 min each. Finally, The H&E slides were sealed with neutral gum and scanned using an iMScope TRIO (Shimadzu, Japan) instrument.

### Statistical Analysis

Data in bar graphs are expressed as mean ± SEM. The Shapiro-Wilcoxon test was used to test the Gaussian distribution of biological parameters. The Student t test was used for comparison between two groups. The Mann-Whitney test was used for variables that were not normally distributed. Correlations were assessed with the Pearson correlation coefficient or as indicated in the graphs. All statistical analysis was performed using SPSS (version 25), considering value of *p* < 0.05 as statistically significant. ROI analysis by using Imaging MS Solution software (version 1.30). The *p* of comparison for ROI analyses was assessed *via* average peak intensities or signals acquired from MS spectra of areas indicated by ROI. Low values of *p* (*p* < 0.05) denote significant differences between average peak intensities or signals of targets within the stipulated ROIs.

## Results and Discussion

Determination of spatial distribution and quantification of BAs in animal models are challenging due to shortage of suitable methods. Here, we adopted a novel iMScope coupled with MALDI-TOF-MS for simultaneously mapping and semi-quantitating the BAs in tissues of mice. There is no precedent to analyze the spatial distribution and quantitative study of BAs within the enterohepatic circulation. Moreover, our work makes it possible to detect the relative content and spatial distribution of BAs in liver, ileum, and colon tissue sections from short-term HFD feeding mice, which provides a new perspective for the study of gastrointestinal diseases.

### The Optimal Laser Intensity for Each Set “Laser Diameter” Value and the Diameter of the Laser in That Case

Supplementary Figure 2 shows approximate values of the optimal laser intensity for each set “Laser Diameter” value and the diameter of the laser in that case. Use this as a guide for the laser intensity and measurement pitch to be set when changing the laser diameter. The example in Supplementary Figure 2 is for the optimal laser intensity when depositing DHB in mouse liver homogenate and measuring lipids in positive ion mode.

### *N*-1-Naphthylphthalamic Acid Was Selected as an Internal Standard to Semi-Quantify Bile Acids in Tissues

As shown in Supplementary Figure 1, the IS (NPA) is seen at *m/z* 290.082, although NPA is chemically different from BAs, there are an adequate signal and no interference peaks in the mass spectrum of NPA. Therefore, it is a feasible method to quantitatively describe the relative exposure level of BAs in tissue sections by the average intensity ratio of each BA to NPA. Two of the major impediments to the application of MALDI to quantitative experiments are signal suppression and signal interference, especially, of less concentrated components (24). However, this effect can be compensated for the use of an IS and optimization of the experimental parameters (24). NPA, chemically different from BAs, has been proved to be effective in quantifying BAs directly from plasma and urine by MALDI-TOF-MS (22, 23). In our study, we compared the BA content of different groups, we sprayed 10 mg ml^–1^ NPA evenly on the tissue sections, and it could be clearly seen through iMScope that NPA exhibits good uniformity and stability. We divided the average peak intensity of BAs by the average peak intensity of NPA to obtain the average peak intensity ratio of each BA. We compared the average peak intensity ratio of each BA in different groups to obtain the final results, which eliminated the influence of signal suppression and signal interference of different groups on the comparison results to a certain extent.

### The Structures of Bile Acids

In our study, eleven BAs conjugates were selected for analysis: DCA, CDCA, UDCA, TDCA, TCDCA, TUDCA, LCA, TLCA, TCA, TMCA, and GCA. The structures for these compounds are shown in Supplementary Figure 3. It should be noted that the putative identification using MSI technology is only based on the measured accurate *m/z* value, so it is impossible to distinguish the isomers of the target compound with the same molecular formula (e.g., *m/z* 498.2895, putative identification as TCDCA/TUDCA/TDCA). BAs show a high degree of isomerism and similar binding (11, 25), as a result, many BAs share the same molecular formula, so it is impossible to determine the contribution of several isomers (e.g., TCDCA, TUDCA, and TDCA) in the net *m/z* signal peak intensity (*m/z* 498.2895 here) and the corresponding *m/z* image. Based on previous quantitative studies of BAs in rat liver, we assumed that all isomers contributed to the ion abundance (25, 26). In order to distinguish the contribution of each isomer to ion abundance, MS^n^ of commercially available standards of the different isomers should be considered.

### 9-Aminoacridine Was Selected as Matrix for the Analysis of Bile Acids Based on Novel Imaging Mass Microscope

The MALDI matrixes, aromatic compounds of low molecular mass, which are crucial for optimal signal-to-noise levels and the quality of data, are used to enhance the ionization efficiency and prevent the analytes from degrading. Owing to the specific ionization property of the individual matrix, the selection of a suitable matrix is understandably critical to MALDI-MS. MALDI matrices are chosen based on their ability to provide sufficient ionization efficiency for a given analytes class (e.g. low molecular weight metabolites, lipids, proteins, polymers, or inorganic compounds) or sub-class (27). Differences in observed analyte sensitivities can be attributed to the physical properties of a matrix such as molecular structure, pH, proton affinity, and peak wavelength absorbance (28). Thus, we investigated three conventional matrices, namely DHB, CHCA and 9AA, to screen the optimum matrix via studying their abilities to form co-crystals onto tissue sections. DHB is the most widely employed and studied MALDI matrix offering sufficient sensitives for many analyte classes in MS analysis (29). 9AA is often used for the analysis of low molecular weight compounds in negative ion mode MS analysis and CHCA ionizes many drugs with high sensitivity in both polarities (30). As shown in Supplementary Figure 4, the ion signal of BAs generated by iMScope is evenly scattered over the tissue sections. The average ion signal intensity of the BA images in Supplementary Figures 4B,C (using CHCA and DHB as the matrix) is much lower than that in Supplementary Figure 4A (using 9AA as the matrix). According to the findings above, 9AA was confirmed to be the most suitable matrix for the analysis of BAs based on iMScope. In conclusion, by using 9AA as the matrix and NPA as IS, we have developed a method for semi-quantitative BAs using MALDI-IMS.

### Mass Spectrometry Imaging-Based Visual Mapping Profiles of Bile Acids Distributed Within Regions of Liver in High-Fat Diet-Fed Mice

**FIGURE 1:**
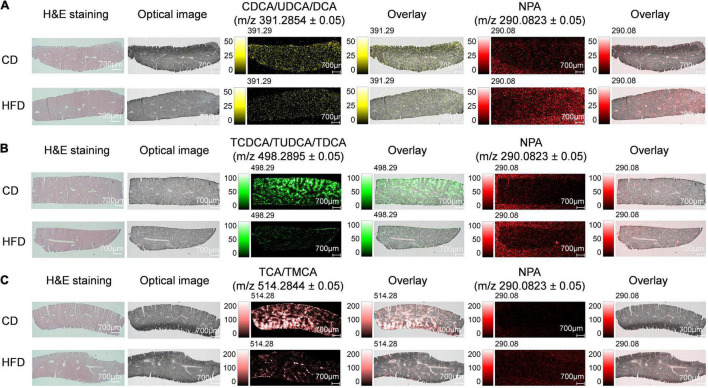
MS imaging-based visual mapping profiles of BAs distributed within regions of liver. MS ion images representing spatial distributions of NPA (*m/z* 290.0823 ± 0.05) and the identified BAs at CDCA/UDCA/DCA *m/z* 391.2854 ± 0.05 **(A)**, TCDCA/TUDCA/TDCA *m/z* 498.2895 ± 0.05 **(B)** and TCA/TMCA *m/z* 514.2844 ± 0.05 **(C)** for the whole section of mouse liver (Scale bars: 1,000 μm). Overlay: Overlay of each MS ion image and optical image. All ion images were normalized to the 9AA matrix signal. Abbreviation: CD, control diet group; HFD, high-fat diet group.

Bile acid synthesis occurs in the liver. Before the synthetic BA enters the duodenum, the liver BAs bind to glycine or taurine. The conjugated BA is stored in the gallbladder until it is released to the duodenum after eating (31). BAs in the human body are mainly conjugated to glycine and less to taurine, while BAs in mice and rats are almost completely conjugated to taurine (6). Here, we detected non-conjugated BAs CDCA/UDCA/DCA and conjugated BAs TCDCA/TUDCA/TDCA and TCA/TMCA in the liver. We found that when compared with the control group MS ion image, the ion intensity of CDCA/UDCA/DCA, TCDCA/TUDCA/TDCA and TCA/TMCA in liver tissue sections of mice fed with HFD for 4 weeks was decreased significantly, especially, TCDCA/TUDCA/TDCA, TCA/TMCA (Figure 1). Supplementary Figures 5A,C,E, respectively, shows the mass spectra of CDCA/UDCA/DCA, TCDCA/TUDCA/TDCA, TCA/TMCA and NPA. NPA-based mean peak intensity ratios also showed that CDCA/UDCA/DCA, TCDCA/TUDCA/TDCA and TCA/TMCA in liver tissue sections of mice fed with HFD for 4 weeks were lower than those in the control group (Supplementary Figures 5B,D,F). BAs have become established as important signaling molecules that enable fine-tuned inter-tissue communication within the enterohepatic circulation. Mounting pieces of evidence indicate that changes in overall BA levels and specific BA metabolites are involved in metabolic diseases, gastrointestinal, and liver cancer (32).

**FIGURE 2:**
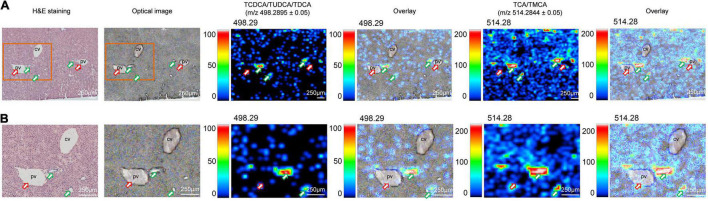
Imaging MS visualize the zonation patterns of BA metabolism in liver of HFD-fed mice. MS ion images representing spatial distributions of the identified taurine-conjugated BAs at *m/z* 498.2895 ± 0.05 (TCDCA/TUDCA/TDCA) and *m/z* 514.2844 ± 0.05 (TCA/TMCA) for the mouse liver in same tissue section at high spatial resolution. Overlay: Overlay of each MS ion image and optical image. All ion images were normalized to the 9AA matrix signal. The green arrows indicated bile ducts, whereas the red arrows showed blood vessels. Scale bars: 250 μm.

Here, we clearly showed that 1 month of HFD-feeding resulted in visible multiple BAs zonation patterns and might have disturbed the inherent BAs gradients or functional zonal differences in enterohepatic circulation. Taking the spatial information into account is crucial to investigate the underlying mechanisms of injury induced by site-specific BA alterations. Combined with the transport network of BAs in the liver, we analyzed the distribution of TCDCA/TUDCA/TDCA and TCA/TMCA in mouse liver tissue sections at high spatial resolution. As expected, BAs were not evenly distributed in the liver and showed a certain regional aggregation pattern, which had been clearly shown from the MS ion image (Figure 2). It should be pointed out that Figure 2 is derived from the HFD group in Figure 1A. The green arrows indicated bile ducts, whereas the red arrows showed blood vessels. Figure 2 shows MS ion images of the spatial distribution of taurine-conjugated BAs identified in the same tissue section at high spatial resolution. The results showed that there were higher ion signal intensities at *m/z* 498.2895 ± 0.05 (TCDCA/TUDCA/TDCA) and *m/z* 514.2844 ± 0.05 (TCA/TMCA) at the bile ducts indicated by the green arrows. The bile duct, portal venule, and portal arteriole form the portal trial, in which the bile duct receives bile from bile canaliculi. Our results have shown that MS ion images in the same liver tissue section at high spatial resolution show that there are zonation patterns and regional differences characteristics of BAs in tissues. Thus, the liver, BAs site of production, displayed physiological and functional zonal differences in the periportal zone versus the centrilobular zone. Therefore, our method can not only explore the spatial distribution characteristics and content variety of BAs in the liver but also intuitively see the distribution characteristics of BAs around blood vessels and bile ducts in liver tissue.

### Mass Spectrometry Imaging-Based Visual Mapping Profiles of Bile Acids Distributed Within Regions of Ileum and Colon in High-Fat Diet-Fed Mice

**FIGURE 3:**
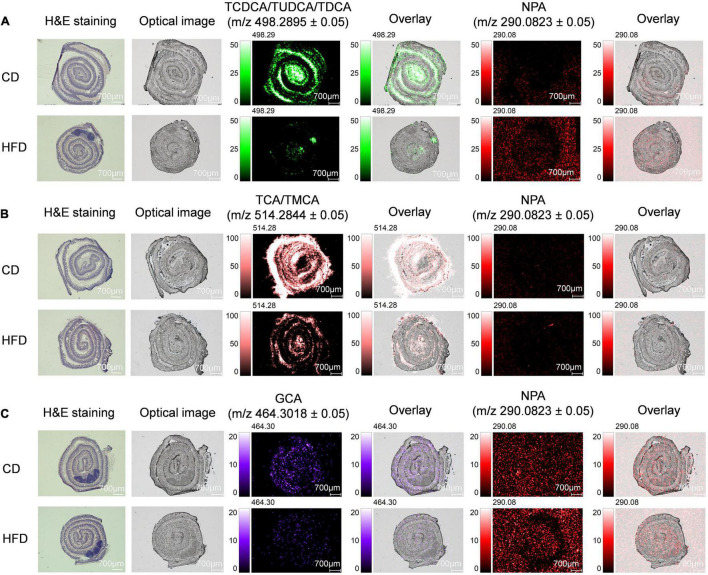
MS imaging-based visual mapping profiles of BAs distributed within regions of ileum. MS ion images representing spatial distributions of NPA (*m/z* 290.0823 ± 0.05) and the identified BAs at TCDCA/TUDCA/TDCA *m/z* 498.2895 ± 0.05 **(A)**, TCA/TMCA *m/z* 514.2844 ± 0.05 **(B)** and GCA *m/z* 464.3018 ± 0.05 **(C)** for the whole section of mouse ileum (Scale bars: 700 μm). Overlay: Overlay of each MS ion image and optical image. All ion images were normalized to the 9AA matrix signal. Abbreviation: CD, control diet group; HFD, high-fat diet group.

The intestinal tract, modified by the gut microbiota, showed regional differences in BA metabolism (33). After exerting physiological functions, such as dissolving and digesting fat-soluble nutrients, most of BAs are then absorbed by passive diffusion and active transport from the terminal ileum and transported back to the liver *via* the portal vein, and the rest is excreted with feces. Therefore, the terminal ileum is also the main site of BAs accumulation, especially, conjugated BAs. We took a 3-cm long mouse terminal ileum and rolled it up in a “Swiss roll” (34). Here, we focused on the level of BAs in the ileum. The spatial distribution of TCDCA/TUDCA/TDCA, TCA/TMCA, GCA, and NPA in ileum sections is shown in Figure 3. We found that when compared with the control group MS ion image, the ion intensity of TCDCA/TUDCA/TDCA, TCA/TMCA, and GCA in ileum tissue sections of mice fed with HFD for 4 weeks was decreased significantly, especially TCDCA/TUDCA/TDCA and TCA/TMCA decreased more significantly, which were also proved by the average peak intensity ratio based on NPA (Supplementary Figures 6B,D,F). TCA/TMCA signal strength was the highest. Supplementary Figures 6A,C,E shows the mass spectra of TCDCA/TUDCA/TDCA, TCA/TMCA, GCA, and NPA.

**FIGURE 4:**
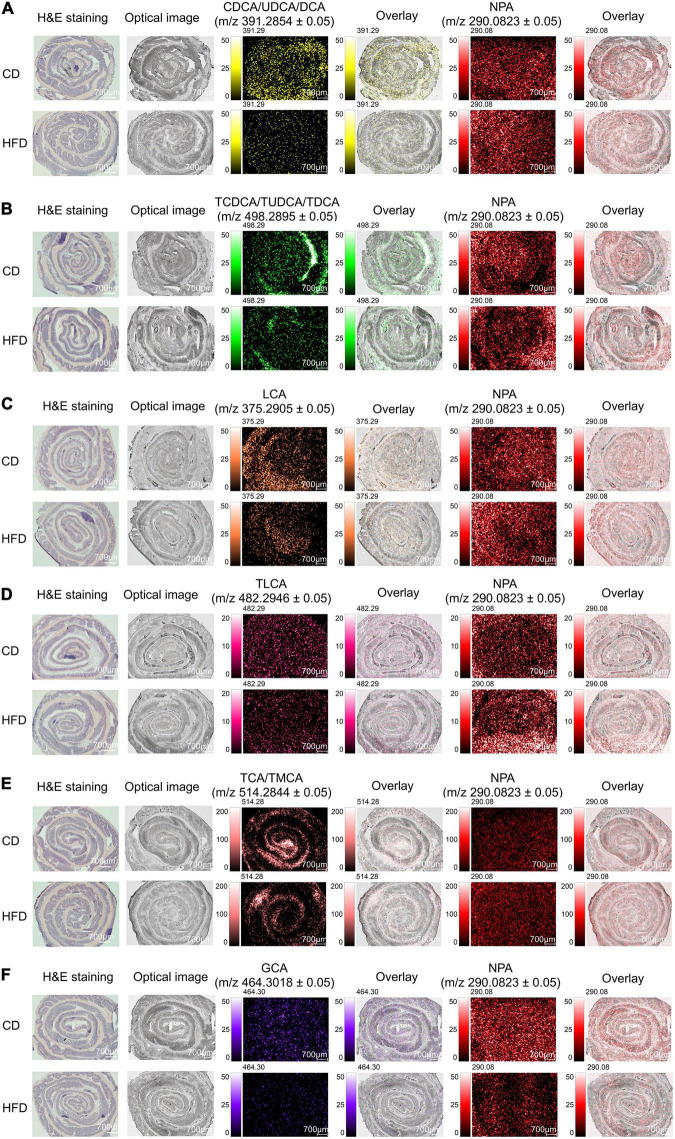
MS imaging-based visual mapping profiles of BAs distributed within regions of colon. MS ion images representing spatial distributions of NPA (*m/z* 290.0823 ± 0.05) and the identified BAs at CDCA/UDCA/DCA *m/z* 391.2854 ± 0.05 **(A)**, TCDCA/TUDCA/TDCA *m/z* 498.2895 ± 0.05 **(B)**, LCA *m/z* 375.2905 ± 0.05 **(C)**, TLCA *m/z* 482.2946 ± 0.05 **(D)**, TCA/TMCA *m/z* 514.2844 ± 0.05 **(E)** and GCA *m/z* 464.3018 ± 0.05 **(F)** for the whole section of mouse colon (Scale bars: 700 μm). Overlay: Overlay of each MS ion image and optical image. All ion images were normalized to the 9AA matrix signal. Abbreviation: CD, control diet group; HFD, high-fat diet group.

In the colon, bacterial enzymes catalyze the deconjugation and dehydroxylation of primary BAs to produce secondary BAs. Here, we focused on the level of BAs in the colon, eleven BA signals are found in the tissue, as shown in Figure 4. All ion images were created based on the signals from the deprotonated molecules: NPA at *m/z* 290.0823 ± 0.05, CDCA/UDCA/DCA at *m/z* 391.2854 ± 0.05, TCDCA/TUDCA/TDCA at *m/z* 498.2895 ± 0.05, LCA at *m/z* 375.2905 ± 0.05, TLCA at *m/z* 482.2946 ± 0.05, TCA/TMCA at *m/z* 514.2844 ± 0.05, and GCA at *m/z* 464.3018 ± 0.05. The grating width of the whole tissue section was 700 μm (Figure 4). The spatial distribution of CDCA/UDCA/DCA and NPA in colon sections is shown in Figure 4A. Supplementary Figure 7A shows the mass spectra of CDCA/UDCA/DCA and NPA. We found that when compared with the control group MS ion image, the ion intensity of CDCA/UDCA/DCA in colonic tissue sections of mice fed with HFD for 4 weeks was decreased significantly, which was also proved by the average peak intensity ratio of CDCA/UDCA/DCA based on NPA (Supplementary Figure 7B). The MS ion images of the other eight observed BAs showed the same tendency as CDCA/UDCA/DCA. After feeding with HFD for 4 weeks, the ion intensities of TCDCA/TUDCA/TDCA, LCA, TLCA, TCA/TMCA, and GCA were decreased significantly (Figures 4B–F). The average peak intensity ratio of each BA based on NPA was also significantly different (Supplementary Figures 7D,F,H,J,L), and the trend was consistent with their MS ion images. Supplementary Figures 7C,E,G,I,K shows the mass spectra of TCDCA/TUDCA/TDCA, LCA, TLCA, TCA/TMCA, GCA, and NPA. By overlaying the acquired MS ion images with their respective optical images (Figure 4), it was discovered that the intensities of BAs were diffusely distributed in colonic tissue. Our results indicate that HFD feeding reduced BAs signal in colon sections by impairing the active and passive reabsorption of BAs in the intestine, which resulted in a significant increase in fecal excretion of BAs. In addition, it is worth noting that BAs play a crucial role in limiting bacterial overgrowth through intestinal FXR, so as to protect the intestine from bacterial damage, and the chronic low-grade inflammation of the intestine caused by HFD may be related to the decrease of BAs content in the intestinal tissue sections observed in this study (1–5).

**FIGURE 5:**
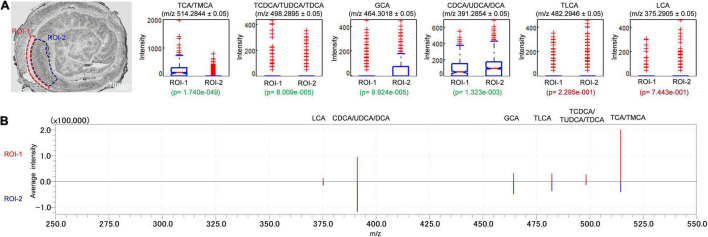
Distribution profiles of BAs in colon were studied through comparison of relative intensities of BAs by the values of p between ROI-1 and ROI-2. Signal intensities of targeted BAs in ROI of two different regions of colon were depicted and signal normalization was performed by pixel. ROI-1: submucosa, muscular layer and adventitia; ROI-2: mucosa layer. The letter ‘*p*’ denotes the statistical *p*-value of the comparison, significant differences were highlighted in green font while insignificant differences were highlighted in red font **(A)**. The spectrum of each ROI which set in **(A)**. The spectra were vertically inverted, with that of ROI-1 above in red and that of ROI-2 below in blue **(B)**.

To evaluate the relative abundance and distribution of detectable BAs in the sampled colon tissues, we further analyzed the mass spectra data through multiple ROI analyses of detectable BAs (Figure 5). Several serial sections (each measuring 10 μm) of sampled colon tissues were used to evaluate the reproducibility of iMScope technique. The relative abundance of BAs by their values of *p* were compared between randomly selected ROIs in submucosa, muscular layer, and adventitia (ROI-1) and mucosa layer (ROI-2) of the sectioned colon tissues. The *p* in this context is defined as the statistical analyses of average intensities/signals acquired from areas specified by ROI. A small *p* (*p* < 0.05) denotes a low likelihood that average intensity values are equal between ROIs. For colonic tissue sections, the relative abundances of TCA/TMCA, TCDCA/TUDCA/TDCA, GCA, and CDCA/UDCA/DCA in the ROI-1 were significantly different from ROI-2 (Figure 5A). The box plot displays the intensity distribution of each measurement point for the applicable *m/z* value. ROI-1 is displayed on the left and ROI-2 on the right. The upper and lower blue lines are displayed at the position of 25% (25th if there are 100 measurement points) and 75%, respectively. The red line in the center of the “box” is the median. The notches of the “box” represent the difference of the median. The notches indicate the 95% confidence interval (CI) of the median (the median range of the population estimated from the median of samples with a probability of 95%). If the 95% CI of median (notch length) does not overlap between two groups (ROI-1 and ROI-2), the two groups are considered different in the median with a probability of 95% (*p* < 0.05). However, overlapping does not always indicate that there is no significant difference between the two groups. In other words, if the notches of ROI-1 and ROI-2 are not at the same height, this means that the intensity of ROI-1 and that of ROI-2 have medians that differ by a significance level of 5%. The “whiskers” extending upward and downward from the “box” indicate the range of 1.5× the length of the box. Intensity values beyond the length of the “whiskers” are displayed with a red+ as outliers with significant deviance from the median. If the median is not in the center of the “box,” this means that the intensity distribution is skewed. When checking the test results, the statistical values of the samples (spectrum peak intensity at measurement points) visualized with box plots can be referred to values of *p*. Figure 5B is the spectrum of each ROI which is set in Figure 5A. The spectra are vertically inverted, with that of ROI-1 above in red and that of ROI-2 below in blue. The abundances of TCA/TMCA and TCDCA/TUDCA/TDCA were higher in the ROI-1, while the relative abundances of GCA and CDCA/UDCA/DCA in the ROI-2 were higher than ROI-1. In addition, there were no significant differences in the distribution of TLCA and LCA in ROI-1 and ROI-2. A significant advantage of the iMScope we used was that we further analyzed the mass spectra data through multiple ROI analyses of detectable BAs. We have identified that the relative abundances of TCA/TMCA, TCDCA/TUDCA/TDCA, GCA, and CDCA/UDCA/DCA in the ROI of the colonic mucosal layer were significantly different from submucosa, muscular layer, and adventitia. Overall, one important future direction of ROI analyses and high spatial resolution is to connect the spatial distribution and signal intensity of BAs with tissue damage in a disease state, so as to provide guidance for us to study the pathogenesis and treatment of gastrointestinal diseases.

Our previous work, and the work of others, showed that only relevant BA changes but not spatial information were obtained since the whole tissue is extracted in one homogenate when traditional MS spectrometry techniques [such as high-performance liquid chromatography (HPLC)-MS/MS] were adopted (2–4, 35–38). However, huge increasing demand on the spatial distribution and content of BAs has become an essential part of metabolic-associated diseases and cancer research (6–10, 32, 33, 39). MALDI-MS can not only analyze and visualize selected molecules but also maintain their spatial distribution and the integrity of the sample, which is an advantage that other MS technologies do not have (40, 41). Genangeli et al. investigated the effect of soyasaponin on the distribution of BAs in duodenum and colon by MALDI-MSI (41). In other study, Kampa et al. (25) investigated the spatial abundances of Amitriptyline, lipids, and BAs in Amitriptyline-treated male rat liver tissue by using MALDI-MSI, and they found two BA signals *m/z* 498.289 (TCDCA/TDCA) and *m/z* 514.284 (TCA/TMCA) showed slight downregulation due to Amitriptyline treatment without comment upon the role of BAs homeostasis in the observed toxicological response. Specifically, the fold change of Bas is based on the average peak intensity of the particular monoisotopic peak within the analyzed region (25). Furthermore, MSI, which combines detailed molecular characterization with spatial distribution measurements, has been applied for determining spatial distribution of BAs in mouse liver biliary networks and also has a certain application in the spatial distribution of BAs in the intestine (11, 25, 41). Although there are important discoveries revealed by these studies, there are also limitations. First, less high-resolution spatial location information; second, without quantitative research combined with spatial location information. Thus, further exploration of BAs on quantitative and interaction with histopathological damage should be examined. iMScope is a powerful tool for MSI, which combines detailed molecular characterization with spatial distribution measurement. Especially the combination of optical microscope and mass spectrometer. The microscope area can be enlarged from 1.25 to 40 times with a CCD camera. In the mass spectrum, the combination of the ion trap and TOF enables it to have both the MS capability of the ion trap and the high-precision mass measurement capability based on TOF, which can achieve excellent sensitivity, good repeatability, and stable mass accuracy (42). Therefore, iMScope is ideal to accurately determine the ROI of the liver and intestines. In addition, the MALDI-MSI technique does not require *a priori* information about the chemical substances present in the tissue section, thus allowing the distribution of many different substances to be mapped in a single experiment (43). Our method, in which 9AA is used as matrix and NPA as IS, can simultaneously measure eleven different BAs by the MALDI-MSI technique. Meanwhile, iMScope can also provide information about the morphology and tissue heterogeneity of specific regions (44, 45). It can increase the applicability of the experimental method.

In summary, in addition to MS ion images that can identify the spatial distribution of BAs with low spatial resolution, the iMScope can also display high spatial resolution MS ion images of the same tissue section. Using a short-term HFD feeding mice model, we fully verified the feasibility and sensitivity of our current method for analyzing the spatial distribution and content of BAs in tissues. Our study further demonstrates even greater potency for a more clear understanding of the mechanism and treatment of gastrointestinal diseases by given spatial distribution and content information of BAs within the enterohepatic circulation.

## Conclusion

Our method can not only detect the relative content of BAs in tissues, but also intuitively display and compare the changes and spatial distribution of BAs content or intensity in different parts of the same tissue. This method has wide application value. Taking the spatial information of BAs into account is crucial to investigate the underlying mechanisms of tissues injury induced by site-specific metabolic alterations, one important future direction of analysis of BAs is fully deciphering the complete molecular signatures of BAs in different biological samples and link them to the pathology of the corresponding diseases. Our method has laid a solid foundation for this development direction.

## Data Availability Statement

The original contributions presented in the study are included in the article/Supplementary Material, further inquiries can be directed to the corresponding authors.

## Ethics Statement

The animal study was reviewed and approved by China Medical University.

## Author Contributions

H-XL and Z-YW conceived of the study and participated in its design and coordination. QZ and Z-HW carried out the experimental work and were responsible for analyzing the data. Z-HW, S-SZ, JY, LC, and X-YW were responsible for animal experiments. All authors have given approval to the final version of the manuscript.

## Conflict of Interest

The authors declare that the research was conducted in the absence of any commercial or financial relationships that could be construed as a potential conflict of interest.

## Publisher’s Note

All claims expressed in this article are solely those of the authors and do not necessarily represent those of their affiliated organizations, or those of the publisher, the editors and the reviewers. Any product that may be evaluated in this article, or claim that may be made by its manufacturer, is not guaranteed or endorsed by the publisher.

## Supplementary Material

Supplementary Figure 1 The structures and mass spectrum of NPA.

Supplementary Figure 2 Approximate values of the optimal laser intensity for each set “laser diameter” value and the diameter of the laser in that case.

Supplementary Figure 3 The structures of BAs.

Supplementary Figure 4 Imaging MS analysis of the BAs in mouse liver generated by iMScope (The scale bar is 700 μm). Imaging MS analysis of TDCA/TCDCA/TUDCA at *m/z* 498.2895 ± 0.05; TCA/TMCA at *m/z* 514.2844 ± 0.05 with 9AA as the matrix **(A)**. Imaging MS analysis of TDCA/TCDCA/TUDCA at *m/z* 498.2895 ± 0.05; TCA/TMCA at *m/z* 514.2844 ± 0.05 with CHCA as the matrix **(B)**. Imaging MS analysis of TDCA/TCDCA/TUDCA at *m/z* 498.2895 ± 0.05; TCA/TMCA at *m/z* 514.2844 ± 0.05 with DHB as the matrix **(C)**. The structures of 9AA, DHB and CHCA **(D)**.

Supplementary Figure 5 MALDI-MSI single pixel mass spectra from liver tissue and average peak intensity ratio of BAs. ^**^*p* < 0.01, ^*⁣**^*p* < 0.001 versus CD group (*n ge* 6).

Supplementary Figure 6 MALDI-MSI single pixel mass spectra from ileum tissue and average peak intensity ratio of BAs. ^**^*p* < 0.01, ^*⁣**^*p* < 0.001 versus CD group (*n ge* 6).

Supplementary Figure 7 MALDI-MSI single pixel mass spectra from colon tissue and average peak intensity ratio of BAs. **p* < 0.05, ^**^*p* < 0.01, ^*⁣**^*p* < 0.001 versus CD group (*n ge* 6).
