# Supplementary figures and images for: Identification and Spatial Visualization of Dysregulated Bile Acid Metabolism in High-Fat Diet-Fed Mice by Mass Spectral Imaging
Source: Front Nutr. 2022 Mar 30;9:858603. doi: 10.3389/fnut.2022.858603 (PMC9007086; doi:10.3389/fnut.2022.858603)

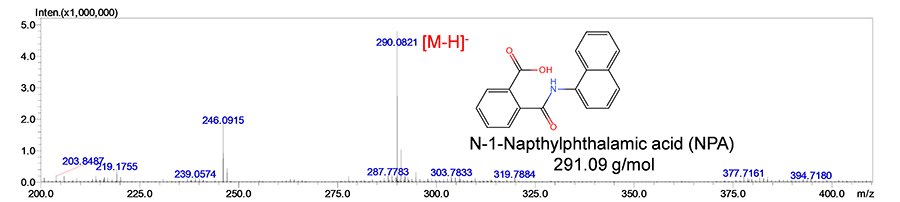

Supplement: Supplementary Figure 1 — The structures and mass spectrum of NPA. [file Image_1.TIF]

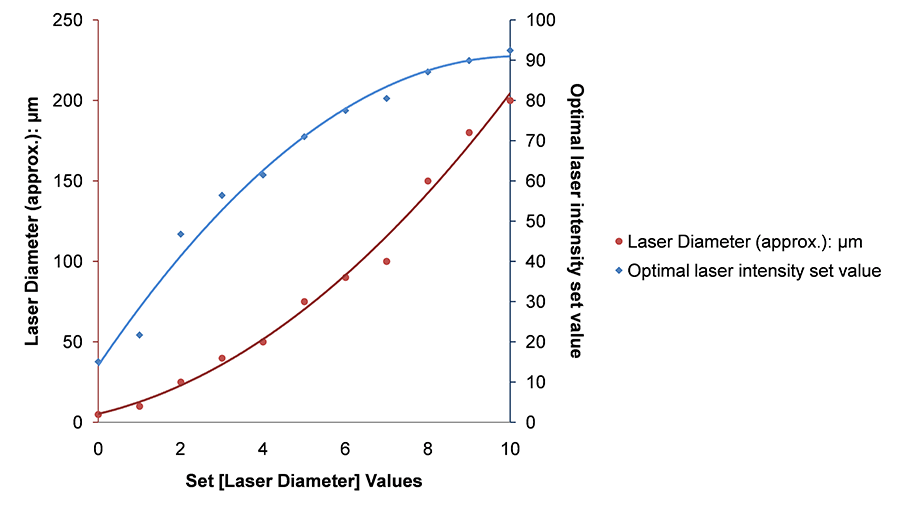

Supplement: Supplementary Figure 2 — Approximate values of the optimal laser intensity for each set “laser diameter” value and the diameter of the laser in that case. [file Image_2.TIF]

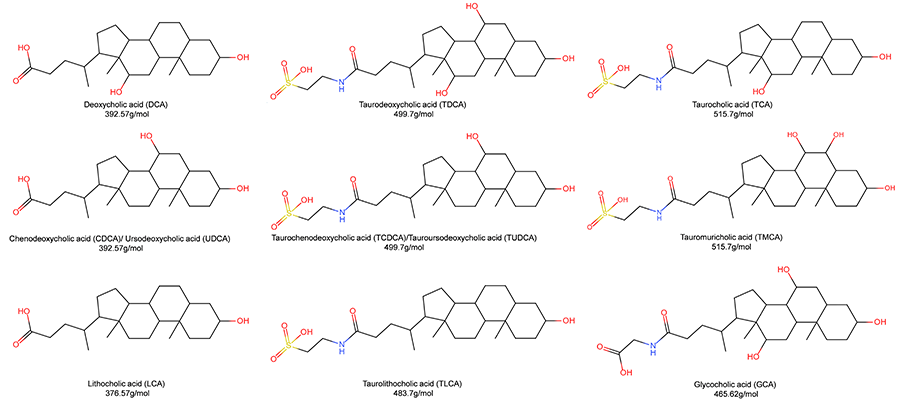

Supplement: Supplementary Figure 3 — The structures of BAs. [file Image_3.TIF]

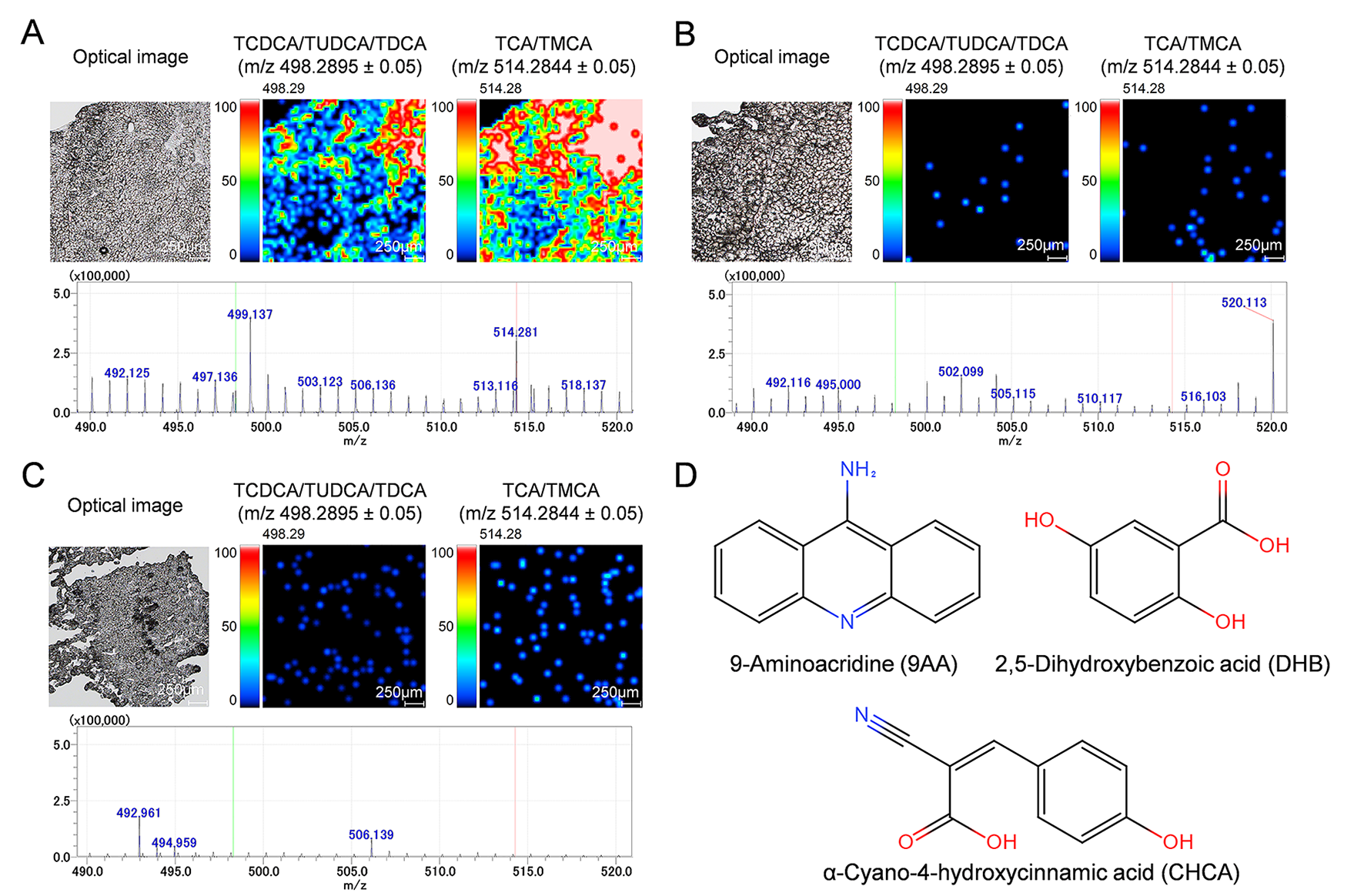

Supplement: Supplementary Figure 4 — Imaging MS analysis of the BAs in mouse liver generated by iMScope (The scale bar is 700 μm). Imaging MS analysis of TDCA/TCDCA/TUDCA at m/z 498.2895 ± 0.05; TCA/TMCA at m/z 514.2844 ± 0.05 with 9AA as the matrix (A). Imaging MS analysis of TDCA/TCDCA/TUDCA at m/z 498.2895 ± 0.05; TCA/TMCA at m/z 514.2844 ± 0.05 with CHCA as the matrix (B). Imaging MS analysis of TDCA/TCDCA/TUDCA at m/z 498.2895 ± 0.05; TCA/TMCA at m/z 514.2844 ± 0.05 with DHB as the matrix (C). The structures of 9AA, DHB and CHCA (D). [file Image_4.TIF]

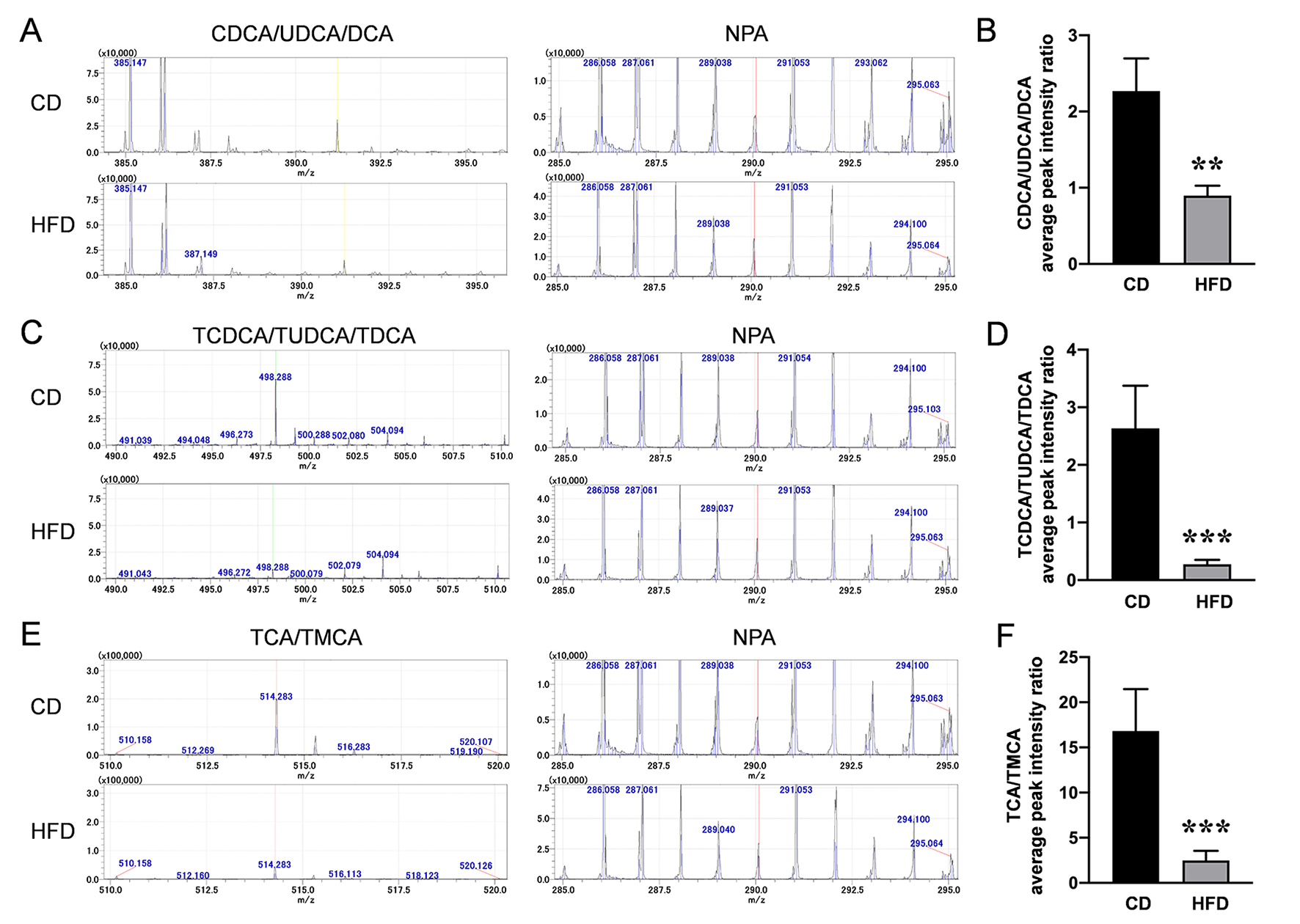

Supplement: Supplementary Figure 5 — MALDI-MSI single pixel mass spectra from liver tissue and average peak intensity ratio of BAs. **p < 0.01, *⁣**p < 0.001 versus CD group (n ge 6). [file Image_5.TIF]

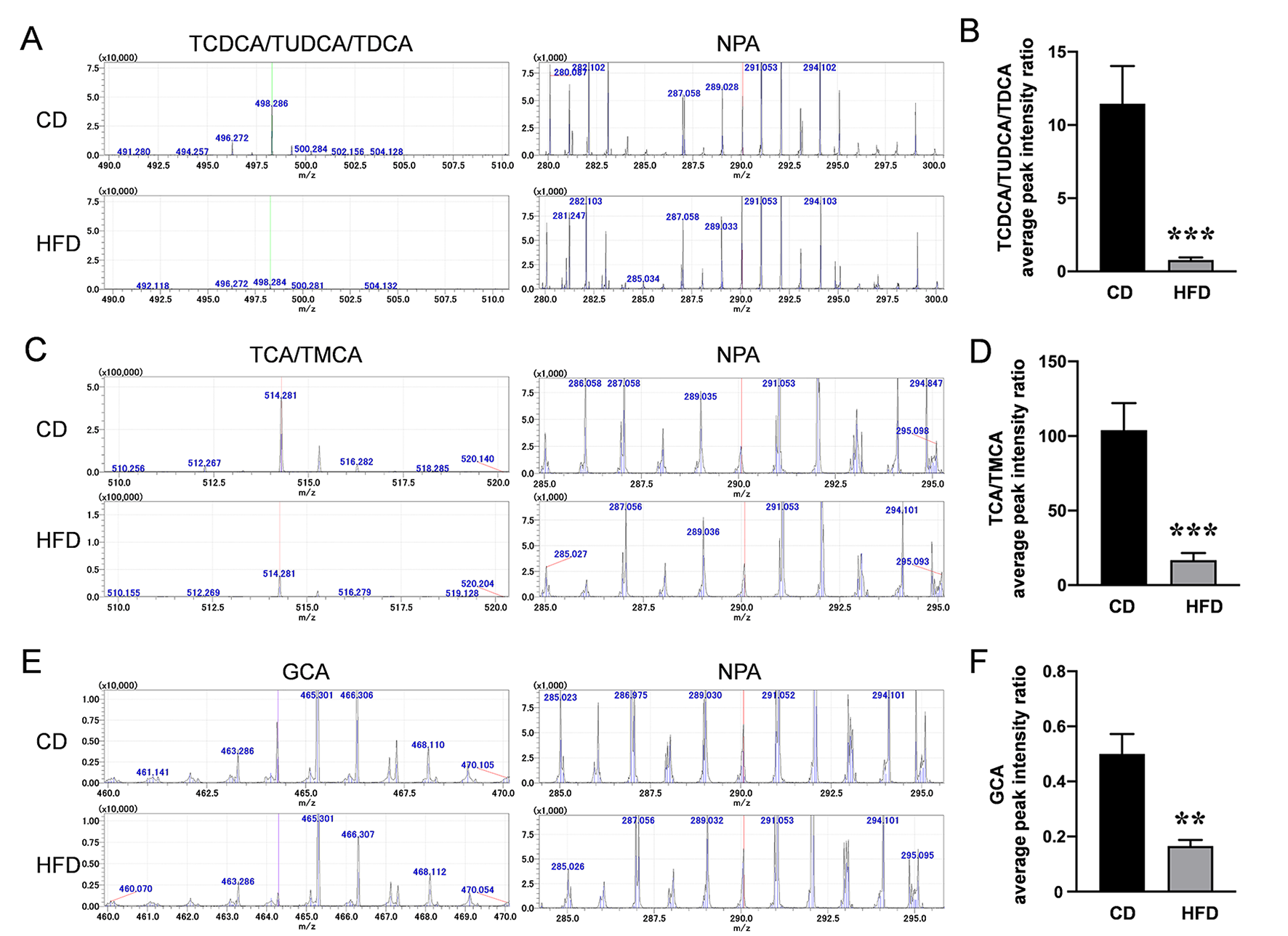

Supplement: Supplementary Figure 6 — MALDI-MSI single pixel mass spectra from ileum tissue and average peak intensity ratio of BAs. **p < 0.01, *⁣**p < 0.001 versus CD group (n ge 6). [file Image_6.TIF]

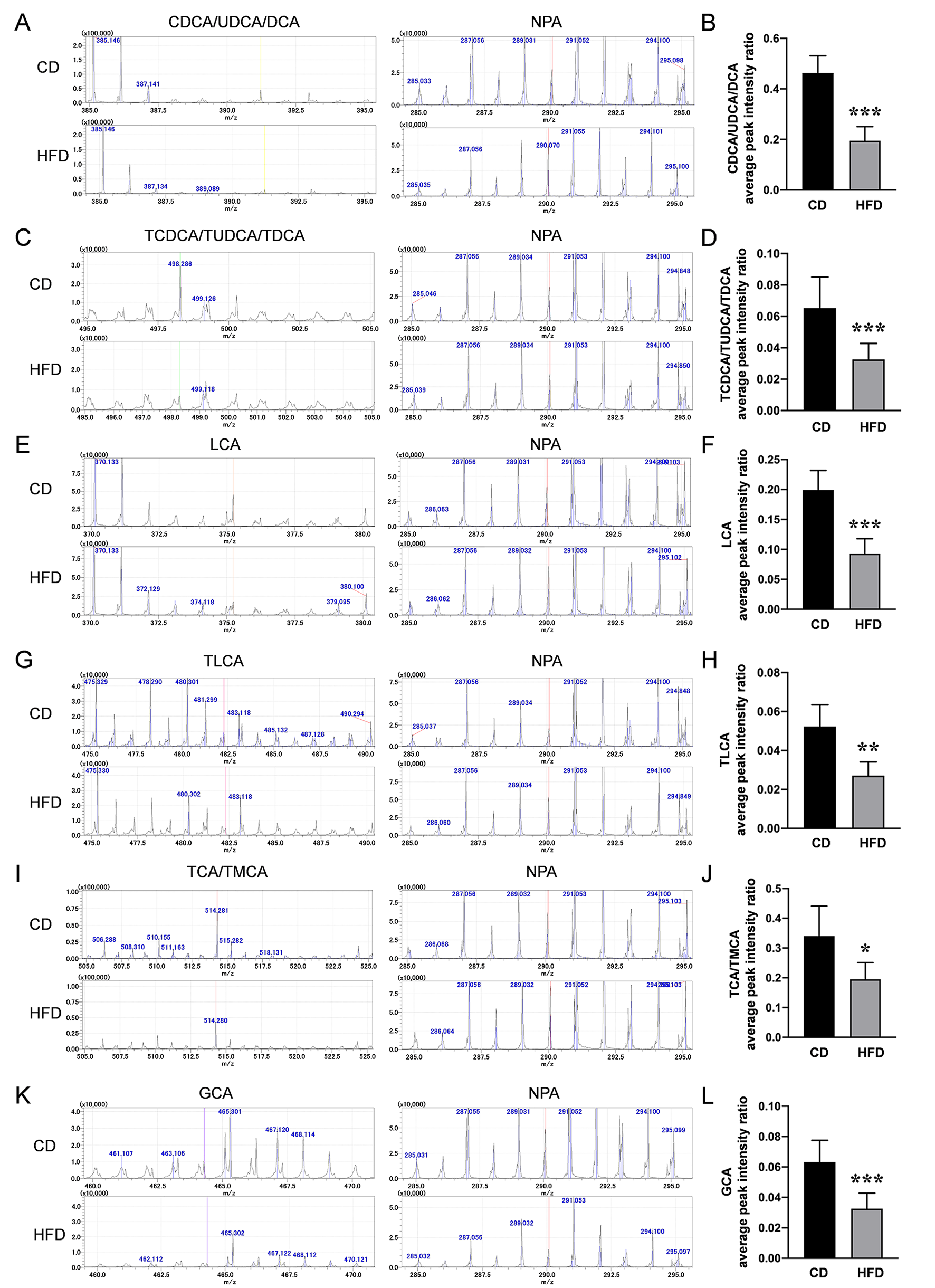

Supplement: Supplementary Figure 7 — MALDI-MSI single pixel mass spectra from colon tissue and average peak intensity ratio of BAs. *p < 0.05, **p < 0.01, *⁣**p < 0.001 versus CD group (n ge 6). [file Image_7.TIF]
